# Extracellular Vesicles in Cell Biology and Medicine

**DOI:** 10.1038/s41598-020-65826-z

**Published:** 2020-05-21

**Authors:** Joana Maria Ramis

**Affiliations:** 1 Cell Therapy and Tissue Engineering Group, Research Institute on Health Sciences (IUNICS), University of the Balearic Islands (UIB), Ctra. Valldemossa km 7.5, 07122 Palma, Spain; 2 Health Research Institute of the Balearic Islands (IdISBa), Palma, Spain; 3 Departament de Biologia Fonamental i Ciències de la Salut, UIB, Palma, Spain

**Keywords:** Medical research, Biomarkers, Cell biology

## Abstract

Extracellular vesicles (EVs) represent a new paradigm, both in cell biology and medicine; specifically, the idea that functional content itself may be delivered directly to cells. EVs are cell-derived membranous structures that work as intercellular communicators exerting their function by transporting their cargo that includes nucleic acids, proteins and lipids. EVs play an essential role in normal physiology, but also in pathological communication, for instance, in cancer, EVs are thought to deliver oncogenic molecules (such as proteins, peptides, RNAs…) to neighboring cells, enhancing propagation of neoplastic cells. Not surprisingly, EV research has become common-place in every field of biomedicine, being explored as diagnostics and therapeutics.

This Collection gathers original Articles that investigate the application of extracellular vesicles on diagnostics and therapeutics, and that report advances in the knowledge of EV biology and the methodological tools for their study.

## Extracellular vesicles in disease

The Articles gathered in this Collection have unveiled a number of ways in which EVs may serve in disease identification, for instance: the EV-associated miRNA of pleural fluids and lavages provide an untapped source of biomarkers for lung cancer diagnosis^1^; chemoresistance in colorectal cancer may be predicted through the evaluation of exosomal circRNA^2^, or exosomal miRNAs may serve for the identification and prognosis of metastatic colorectal cancer^3^; and a decrease in the Gelsolin content of plasma-EVs acts as a biomarker for dementia with Lewy Bodies, distinguishing these patients from those with Alzheimer’s Disease^4^.

Other Articles included in the Collection have explored the use of EVs in therapeutics. For the treatment of immune-related diseases, the application of an inflammatory stimulus is shown to improve the anti-inflammatory and/or immunosuppressive potential of EVs secreted by adipose mesenchymal stem cells^5^. Cytoprotection of stressed cardiomyocytes through the use of EVs derived from mesenchymal stromal cells^6^, and the prevention of glucocorticoid-induced osteoporosis – through the suppression of osteoblasts’ ferroptic pathway – by EVs extracted from bone marrow-derived endothelial progenitor^7^, were also demonstrated. As well as applying naturally occurring EVs, one of the published original Articles has demonstrated engineered EVs as a feasible therapeutic tool. Do *et al*. have developed a chimeric protein by fusing human lysosomal β-glucocerebrosidase (GBA) to an exosome-anchoring protein; this chimeric protein was successfully secreted into EVs, and delivered to recipient cells, providing a potential strategy for the treatment of lysosomal storage diseases^8^.

## Extracellular vesicles’ biology and endogenous function

The Collection also advances our knowledge of EVs biology and function. For example, two articles have studied the lipidome and glycans of EVs, highlighting the lipidome profile as a possible marker to discriminate exosomes from microvesicles^9^, and identifying glycans as key players in the tuning of EV uptake through charge-based effects, direct glycan recognition or both^10^. Other roles of EVs have also been explored, such as oviductal EVs modulating sperm function and fertilization^11^, or EVs from aged astrocytes inhibiting the maturation of oligodendrocyte progenitor cells into oligodendrocytes^12^. In addition, the involvement of EVs in the formation of the pre-metastatic niche^13^, or the neuroprotective role of EVs containing Cystatin C^14^, were also investigated.

## Tools and methods for studying Extracellular Vesicles

Given EVs size and heterogeneity, their isolation, detection and characterization still remains a challenge, although much effort is being made to improve methodological tools for EV study. A new aqueous two-phase system-based isolation protocol for EVs isolation at high efficiency and purity was reported^15^. A further Article reported a new EV immunolabeling method that can be incorporated into existing NTA protocols to provide particle concentration, size distribution, and surface phenotype of EV samples^16^. In addition, a luminescence-based assay for EV uptake assays – clearly discriminating between EV uptake, and EV binding to the target cell – has been developed^17^. Finally, an inducible CD9-GFP mouse was generated, providing a tool that enables EV labelling in a cell-type specific manner, while simultaneously allowing *in vivo* experimentation^18^.

Many thanks to all the contributors (authors and reviewers) to this Collection.
